# Characterization of the Emerging Enteropathogen *Escherichia Albertii* Isolated from Urine Samples of Patients Attending Sapporo Area Hospitals, Japan

**DOI:** 10.1155/2022/4236054

**Published:** 2022-09-16

**Authors:** Miyuki Fujioka, Sho Yoshioka, Masahiko Ito, Chowdhury Rafiqul Ahsan

**Affiliations:** ^1^Hirosaki University Graduate School of Health Sciences, Honcho 66-1, Hirosaki, Aomori 036-8564, Japan; ^2^Sapporo Clinical Laboratory, Kita Sanjo Nishi18-2-2, Sapporo, Hokkaido 0600005, Japan; ^3^Department of Microbiology, University of Dhaka, Dhaka 1000, Bangladesh

## Abstract

Recently, *Escherichia albertii* has been identified as a causative agent of diarrhea in humans and is often misidentified as diarrheagenic *Escherichia coli* (DEC), a lactose-nondegrading bacterium. In this study, we performed biochemical characterization, gene possession status, drug susceptibility testing, and sequencing analysis of the strains detected in urine samples. One urea-degrading strain was detected in terms of biochemical characteristics, but was found to be nonurea-degrading by another method, leading to conflicting results. All target strains possessed the *E. albertii*-specific gene, the DEC common gene *eae*, and the *E. coli* 16S rRNA gene. In the drug susceptibility test, all urine-derived strains were sensitive to tetracycline (TC), whereas the JCM 17328 strain was resistant to TC, suggesting that TC is effective against urine-derived *E. albertii* strains. In 16S rRNA sequencing analysis, the *E. albertii* strains were ranked at the top of homology, but not in the top one, making it difficult to differentiate them from other strains. In summary, if a suspected lactose-nondegrading *E. coli* strain was isolated from a urine sample, it could be differentiated from *E. albertii* by the presence of *E. albertii*-specific genes.

## 1. Introduction

Humans and numerous bird species are susceptible to *Escherichia albertii*, a developing enteropathogen responsible for epidemic mortality [1, 2]. Because of their phenotypic and genetic similarities, such as numerous biochemical traits and the possession of a type III secretion system encoded by the locus of enterocyte effacement, this bacterium has been commonly misdiagnosed as enteropathogenic or enterohemorrhagic *E. coli* [3]. Some *E. coli* strains have acquired virulence genes that confer pathogenicity and are referred to as diarrheagenic *E. coli* (DEC) [4]. However, in general, *E. coli* that cause urinary tract infections (UTI) are said to be different from those that cause diarrhea [5] and there have been reports of DEC detection in the urine [6].

*E. albertii* has biochemical properties similar to those of atypical *E. coli*, making it difficult to identify the bacterial species based on their biochemical properties alone [7, 8]. Since *E. albertii* may be misidentified by mass spectrometry, therefore, the detection of specific genes via PCR is the practical method for identifying *E. albertii* [9]. In recent years, we reported the isolation of *E. albertii* from fecal and urine samples [10]. However, in the present study, *E. albertii* strains isolated from urine samples were characterized in details through biochemical, gene possession status, drug susceptibility testing, and sequencing analysis. It was suggested that *E. albertii* could be identified by searching for the *E. albertii*-specific genes like *mdh*, *lysP*, and *clpX*.

## 2. Materials and Methods

A total of 48,516 bacterial strains were isolated from patients with fecal and urinary tract infections attending different private hospitals around the Sapporo area in Japan. Among the 35,646 urine-derived strains identified as *E. coli* by matrix-assisted laser desorption/ionization time-of-flight mass spectrometry(MALDI-TOF MS, MALDI Biotyper; Bruker, Billerica, MA, USA) and MBT Compass Library (ver. 8.0.0, Bruker), we used six strains that were ranked in the top 10 as *E. albertii* [10]. Note that fully unlinkable anonymized strains were used in this study.

### 2.1. Biochemical Properties

Biochemical properties of *E. albertii* were examined using the API 20E system (bioMérieux, Marcy-l'Étoile, France). Glycolysis tests, which were not performed on the API 20E system, were performed by adding xylose to the BTB lactose agar plates (Nissui, Tokyo, Japan) at a rate of 1% *E. albertii* JCM 17328 was used as the reference strain. The strains those were positive for the urea degradation tests using the API 20E system, were confirmed to be degraded by rapid urea broth (BD, East Rutherford, NJ, USA).

### 2.2. PCR Amplification

Six strains were examined for their genetic characteristics. We amplified the malate dehydrogenase gene (*mdh*), lysine-specific transporter gene (*lysP*) [9], and heat-shock protein gene (*clpX*) [2] as specific genes for *E. albertii* by multiplex PCR. In addition, we used the common gene *eae* between *E. albertii* and DEC, and the *E. coli* 16S rRNA gene for PCR. Oligonucleotide primers used in this study are listed in Table 1.

For DNA extraction, each strain was cultured in 3 mL of Luria–Bertani broth (Sigma–Aldrich Japan, Tokyo, Japan) and incubated overnight at 37°C with shaking at 115 rpm. A 100 *µ*L aliquot of bacterial culture was suspended in 900 *µ*L of Tris-EDTA buffer (Nippon gene, Tokyo, Japan), boiled for 5 min, centrifuged at 10,000 × *g* for 5 min, and the supernatant was collected.

The multiplex PCR was performed as follows: each 25 *µ*L of the reaction mixture contained 20 mM Tris-HCl, 100 mM KCl, 1.5–2.0 mM MgCl2, 2.5 mM of each deoxynucleoside triphosphate(dNTP) mixture, 1.25 U of Taq DNA polymerase (TaKaRa Bio Inc., Shiga, Japan), 25 *µ*M of each primer, and 2.5 *µ*L of the DNA template. These samples, which had been preheated at 94°C for 1 min, were amplified for 25 cycles using a thermal cycler (TaKaRa PCR Thermal Cycler Dice touch, TaKaRa, Shiga, Japan). Each cycle consisted of denaturation at 94°C for 1 min, annealing at 55°C for 1 min, and extension at 72°C for 1 min. The final extension step was performed at 72°C for 10 min. PCR products were separated by electrophoresis on a 2.5% agarose gel.

### 2.3. Antimicrobial Susceptibility Tests

Antimicrobial susceptibility was determined using the Kirby–Bauer disk diffusion method [13] on Mueller–Hinton agar plates (Eiken, Tochigi, Japan). The antibiotics used in this study were 5 *µ*g ofloxacin (OFLX), 5 *µ*g ciprofloxacin (CPFX), 5 *µ*g erythromycin (EM), 1 *µ*g fosfomycin (FOM), and 30 *µ*g tetracycline (TC) (Sensi disk, BD Fukushima, Japan). The inoculated plates were incubated for 24 h aerobically at 37°C. The diameters of the zones of inhibition were interpreted according to the Clinical Laboratory Standards Institute guidelines [14].

### 2.4. 16S rRNA Sequence Analysis

Direct DNA sequencing was performed to accurately identify the six *E. albertii* strains. The template DNA was prepared by suspending one colony of the target *E. albertii*, which had been purely cultured on heart infusion agar medium (Eikenn, Tochigi, Japan) in 1 mL of TE buffer, heating at 100°C for 5 min, followed by centrifugation at 10,000 rpm for 5 min, and collecting the supernatant. The following primers were used for PCR and sequencing; 8UA:5′-AGAGTTTGATC(A/C)TGGCTCAG-3′, 519A:5′-CAGC(A/C)GCCGCGGTAAT-3′, 519B:5′-ATTACCGCGGC(T/G)GCTG-3′, 907A:5′-AAACT(T/C)AAA(T/G)GAATTGACGG-3′, 907B:5′-CCGTCAATTC(A/C)TTT(A/G)AGTTT-3′, and 1485B:5′-TACGGTTACCTTGTTACGAC-3′ [15]. The PCR assay was performed as follows: each 50 µL reaction mixture contained 25 *µ*L of PrimeSTAR polymerase (TaKaRa, Shiga, Japan), 10 *µ*M of each primer, and 1 *µ*L of the DNA template. Each cycle consisted of denaturation at 94°C for 15 sec, annealing at 55°C for 5 sec, and extension at 72°C for 10 sec were amplified for 30 cycles using a thermal cycler. PCR products were separated by electrophoresis on a 2% agarose gel. After confirming the bands by electrophoresis, DNA was extracted from agarose gels for samples with extra bands and from PCR reaction solution for samples without extra bands (gel PCR extraction kit, Nippon Gene, Tokyo, Japan). The purified DNA and the four 9.6 *μ*M sequence primers [15] were subject to 16S rRNA sequence analysis by an outside supplier(Eurofins Genomics, Tokyo, Japan). The obtained sequences were done pairwise comparison by MEGA X software [16] and EZBioCloud [17].

## 3. Results

### 3.1. Biochemical Properties

The biochemical properties of the six *E. albertii* strains are listed in Table 2. All *E. albertii* strains were nonmotile and positive for lysine decarboxylase, ornithine decarboxylase, indole, glucose, mannitol, and L-arabinose, while being negative for arginine dihydrolase, citrate, H_2_S, tryptophan deaminase, Voges–Proskauer, gelatinase, inositol, L-rhamnose, D-melibiose, amygdalin, lactose, and D-xylose. Furthermore, three ONPG-positive *E. albertii* strains, one urea-positive strain, four D-sorbitol-positive strains, and one sucrose-positive strain were detected. In addition, the strain that was positive for urea degradation with API 20E was negative in the rapid urea broth. The six *E. albertii* strains and the JCM17328 strain were identified as *E. coli* using the API system. There were five different combinations of test results from the six isolates, so isolate no. 1 and no. 2 strains have the same results.

### 3.2. PCR Amplification

The results showed that all six *E. albertii* strains and the *E. albertii* JCM 17348 strain harbored *E. albertii-*specifi*c* genes *mdh*, *lysP*, and *clpX* along with the *eae* and *E. coli* 16S rRNA genes.

### 3.3. Antimicrobial Susceptibility Tests

The antimicrobial susceptibility of the *E. albertii* strains to five antibiotics was investigated. Six strains were susceptible to OFLX, CPFX, FOM, and TC but were resistant to EM. The JCM 17328 strain was susceptible to OFLX, CPLX, and FOM but was resistant to TC and EM.

### 3.4. 16S rRNA Sequence Analysis

The sequence homologies with *E. albertii* JCM 17328 is shown in Table 3. Four out of the six strains had the highest homology with *E. albertii*, ranging from 99.44% to 99.52%. One strain had the highest homology with *E. marmotae* (98.92%) and the fifth-highest homology with *E. albertii* (98.41%). The other strain was *Shigella dysenteriae*, with the highest homology (98.97%), followed by *E. albertii* (98.70%). The four strains with the highest homology to *E. albertii* included *E. fergusonii* and *Shigella* among the top five homologs. One strain with the highest homology to *E. marmotae* and one strain with the highest homology to *S. dysenteriae* included not only *E. albertii* but also *E. fergusonii* and *Shigella* in the top five homologous strains. Five different sequences were analyzed from the six strains, and no. 4 and no. 5 strains have the same sequence analysis.

## 4. Discussion

*E. albertii* was first isolated from pediatric stools in Bangladesh in 1991 and was initially identified as *Hafnia alvei* [18]. Subsequently, Huys et al. [1] registered the strain as a new species of the genus *Escherichia* by 16S rRNA sequencing analysis, DNA-DNA hybridization and property analysis. There have been reports of infection cases worldwide, including in Japan [19, 20], but all cases have been isolated from diarrhea-derived stools; there have been no reports of isolation cases from urine samples. In the present study, detailed biochemical properties of the *E. albertii* strain isolated from specimens derived from patients with UTIs were analyzed. Nataro et al. [21] classified *E. albertii* into biotypes 1 and 2 using lysine decarboxylation and the indole reaction. The JCM 17328 strain used in this study was classified as biotype 1 based on lysine decarboxylation positivity and indole reaction negativity. However, six urine-derived strains were positive for lysine decarboxylation but not for the indole reaction and were not classified as either biotype 1 or 2. It was suggested that different types of strains from the JCM 17328 strain derived from diarrheal patients may be the cause of community-acquired UTI. In addition, one urea-degradation-positive strain was detected in API 20E. *E. albertii* is known to be negative for urea degradation [22] and no positive strains have been reported to date. Most *E. coli* strains, which have biochemical properties similar to those of *E. albertii*, are negative for urea decomposition. However, there have been studies using other urea decomposition tests that have found urea decomposition-positive *E. coli* strains [23]. Therefore, it was suggested that different results would be obtained if different urea decomposition test methods were used.

In the drug susceptibility test, the susceptibility of OFLX and CPFX to the new quinolones was good. The antibacterial spectrum of new quinolones is broad and they are indicative of systemic infections, such as pneumonia and UTIs [24]. As such, we believe that they are effective against UTIs caused by *E. albertii*. Stock et al. [25] reported that *E. albertii* is sensitive to FOM, which we have corroborated here, indicating that FOM is effective against *E. albertii* infection. Macrolide EM is a hydrophobic drug with a high molecular weight that does not pass through the hydrophilic porin pores formed in the outer membrane of Gram-negative rods and is considered to have little antibacterial activity against Gram-negative rods but is a known treatment for *Campylobacter* enteritis [26]. *E. albertii* and its close relatives, *E. coli* and *Salmonella*, are considered naturally resistant to EM [27], and *E. albertii* is also EM-resistant. In the present study, the six target strains and JCM17328 strains were EM-resistant, confirming that EM is ineffective against *E. albertii*. Chopra and Roberts [28] reported that Tetracyclines are broad-spectrum agents, exhibiting activity against a wide range of Gram-positive and Gram-negative bacteria, atypical organisms such as *Chlamydia*, *Mycoplasma*, and *Rickettsia*, and protozoan parasites, *E. albertii* is naturally resistant to TC as well as EM. In this study, JCM strains were TC-resistant, but all six strains were TC-sensitive, suggesting that TC is effective against urine-derived strains.

The intimin gene *eae* is known to cause attaching and effacing (A/E) lesions in intestinal tissues [29]. *eae* possessed by enterohaemorrhagic and enteropathogenic *E. coli* (EHEC and EPEC, respectively), which are classified as DECs, is known to be a common gene in *E. albertii* [7]. As such, *E. albertii* may be misidentified as EHEC or EPEC in DEC searches of diarrheal-derived strains. However, DEC-related genes have not been identified in strains suspected to be *E. coli* in urine specimen-derived strains, suggesting that *E. albertii* may have been misidentified as lactose-nondegrading atypical *E. coli*. Abberton et al. [30] reported that the *E. coli* 16S rRNA gene enabled differentiation between *E. coli* and *Shigella*. We tested whether it is possible to distinguish *E. albertii* from *E. coli* by targeting the *E. coli* 16S rRNA gene. All strains, including the JCM17328 strain, harbored the *E. coli* 16S rRNA gene, suggesting that a search for this gene also misidentified the strain as *E. coli*. Currently, *E. albertii* is identified by PCR targeting specific genes: *mdh*, *lysP*, and *clpX* [31–33]. The six *E. albertii* strains in this study possessed these specific genes and were identified as *E. albertii*. Therefore, *eae-*harboring strains need to be differentiated from *E. albertii*, and targeting the specific genes *mdh*, *lysP*, and *clpX* is effective.

Generally, 16S rRNA sequencing analysis is performed for bacteria that are difficult to culture or cannot be differentiated by conventional tests [34] and as such, we differentiated *E. albertii* from the 16S rRNA region. In the present study, we analyzed these strains using direct sequencing, which is used for rapid species identification in clinical laboratories [15]. One strain showed a high homology with *E. marmotae*, which was isolated from guinea pigs living on the Tibetan plateau and was registered as a new species in 2015 [35]. However, there have been no reports to date of infections caused by this organism. *E. marmotae* is characterized by ornithine decarboxylase negativity, rhamnose, and xylose degradation [35]. The strain in the current study did not possess any of these characteristics and was distinguishable from *E. marmotae* because it contained a specific gene for *E. albertii.* In addition, strains with high homology to *S. dysenteriae* could also be differentiated by possession of these specific genes and *E. coli* 16S rRNA genes, and all strains could be identified as *E. albertii*. The 16S rRNA sequence is similar in *Escherichia* species [36], which makes differentiation difficult. Of the strains included in this study, *E. fergusonii* was included within the top five of the six *E. albertii* strains. *E. fergusonii* has been associated with only a few case reports of disease in individuals of animal or human origin [37]. Therefore, it is difficult to identify all *E. albertii* by homology alone in 16S rRNA analysis, and differentiation from *E. fergusonii* is required; however, identification is possible by searching for *E. albertii*-specific genes.

In this study, the two strains showed the same pattern in biochemical properties, but another two strains showed the same pattern in sequence analysis. Even though the properties were the same, the sequence analysis yielded different results, suggesting that *E. albertii* does not show a relationship between biochemical properties and sequence analysis. We believe this makes identification more difficult.

## 5. Conclusion

Diarrhea-causing *E. albertii* was isolated from urine samples. *E. albertii* is difficult to identify using biochemical characterization or 16S rRNA sequencing. To identify *E. albertii*, it is necessary to differentiate it from EHEC and EPEC when the DEC-related gene *eae* is detected in stool samples. In addition, when strains suspected of being lactose-nondegrading atypical *E. coli* were detected in urine samples, it was suggested that *E. albertii* could be identified by searching for the *E. albertii*-specific genes *mdh*, *lysP*, and *clpX*.

## Figures and Tables

**Table 1:** PCR primers used in this study.

Target genes		Sequences (5′ to 3′)	Sizes of PCR product (bp)	Reference
*Escherichia albertii*	mdh	CTGGAAGGCGCAGATGTGGTACTGATT	115	[9]
		CTTGCTGAACCAGATTCTTCACAATACCG		
	lysP	GGGCGCTGCTTTCATATATTCTT	252	[9]
		TCCAGATCCAACCGGGAGTATCAGGA		
	cplX	TGGCGTCGAGTTGGGCA	384	[2]
		TCCTGCTGCGGATGTTTACG		

Diarrheagenic *E. coli*	eae	TGGCGTCGAGTTGGGCA	881	[11]
		TCCTGCTGCGGATGTTTACG		
*Escherichia coli*	16S rRNA	GGAAGAAGCTTGCTTCTTTGCTGA	544	[12]
		AGGCCCGGGGATTTCACATCTGACTT		

**Table 2:** Biochemical properties of *E. albertii* strains.

Agents or tests	*E. albertii* strains isolated from urine samples						Reactions for type strain *E. albertii*
	No. 1	No. 2	No. 3	No. 4	No. 5	No. 6	JCM17328
Motility	－	－	－	－	－	－	－
ONPG	－	－	+	+	+	－	+
Arginine dihydrolase	－	－	－	－	－	－	－
Lysine decarboxylase	+	+	+	+	+	+	+
Ornitine decarboxylase	+	+	+	+	+	+	+
Citrate	－	－	－	－	－	－	－
H2S	－	－	－	－	－	－	－
Urea (API 20E/urea broth)	－	－	－	－	－	+/-†	－
Tryptophan deaminase	－	－	－	－	－	－	－
Indole	+	+	+	+	+	+	－
Voges proskauer	－	－	－	－	－	－	－
Gelatinase	－	－	－	－	－	－	－
Acid from glucose	+	+	+	+	+	+	+
D-mannitol	+	+	+	+	+	+	+
Inositol	－	－	－	－	－	－	－
D-sorbitol	+	+	+	－	－	+	－
L-rhamnose	－	－	－	－	－	－	－
Sucrose	－	－	－	－	+	－	－
D-melibiose	－	－	－	－	－	－	－
D-amygdalin	－	－	－	－	－	－	－
L-arabinose	+	+	+	+	+	+	+
Lactose	－	－	－	－	－	－	－
D-xylose	－	－	－	－	－	－	－

+, positive; −, negative. †: API 20E, positive; rapid urea broth, negative.

**Table 3:** 16S rRNA sequence analysis.

Hit taxon name (hit strain name)	Strain no. (%)				
	1	2	3	4	5
*Escherichia albertii* (TW07627)	**99.22**	98.41	98.70	**99.45**	**99.45**

*Escherichia marmotae* (HT073016)		**98.92**	98.63		

*Escherichia fergusonii* (ATCC35469)	98.96	98.56	98.43	99.04	99.04

*Shigella flexnari* (ATCC29903)	98.88	98.48	98.36	98.98	98.98

*Shigella boydii* (GTC779)	98.80	98.41		98.91	98.91

*Shigella sonnei* (CECT4887)	98.72			98.84	98.84

*Shigella dysenteriae* (ATCC13313)		98.48	**98.97**		

Noted: bold number is the highest homology in each *E. albertii* strain.

## Data Availability

The data used in this study are available from the corresponding author on request.

## List of abbreviations

DNA: Deoxyribonucleic acid
EDTA: Ethylenediaminetetraacetic acid
JCM: Japan collection of microorganisms
ONPG: o-nitrophenyl-*β*-D-galactopyranoside
PCR: Polymerase chain reaction
rRNA: Ribosomal ribonucleic acid.

## Supplementary Materials

Supplementary Materials Supplementary Materials: the results of antimicrobial susceptibility tests are shown in Table S1.
