# Supplementary material for: Characterization of the Emerging Enteropathogen Escherichia Albertii Isolated from Urine Samples of Patients Attending Sapporo Area Hospitals, Japan
Source: Int J Microbiol. 2022 Sep 16;2022:4236054. doi: 10.1155/2022/4236054 (PMC9507761; doi:10.1155/2022/4236054)
Supplement: Supplementary Materials — the results of antimicrobial susceptibility tests are shown in Table S1. [file 4236054.f1.docx]

| **Supplemental material, T A B L E S1**  The measured value of antimicrobial susceptibility tests (mm) | | | | | | | | |
| --- | --- | --- | --- | --- | --- | --- | --- | --- |
| **Strains** | **EM** | **FOM** | **OFLX** | **CPFX** | **TC** |  |  |  |
| No. 1 | 12 | 26 | 34 | 34 | 24 |  |  |  |
| No. 2 | 8 | 30 | 18 | 30 | 22 |  |  |  |
| No. 3 | 9 | 28 | 28 | 31 | 23 |  |  |  |
| No. 4 | 11 | 31 | 31 | 32 | 23 |  |  |  |
| No. 5 | 12 | 30 | 34 | 34 | 25 |  |  |  |
| No. 6 | 9 | 28 | 28 | 31 | 24 |  |  |  |
| *Escherichia albertii* | 8 | 40 | 26 | 30 | 8 |  |  |  |
| JCM 17328 |  |  |  |  |  |  |  |  |
|  | | | | | |  |  |  |
